# Cross-Generational Integration of Exercise and Nutritional Encoding in Offspring Adipose Genomics

**DOI:** 10.3390/ijms27062623

**Published:** 2026-03-13

**Authors:** Song Ah Chae, Choongsung Yoo, Jun Seok Son

**Affiliations:** 1 Laboratory of Perinatal Exercise Genomics, Department of Obstetrics, Gynecology and Reproductive Sciences, University of Maryland School of Medicine, Baltimore, MD 21201, USA; songah.chae@som.umaryland.edu (S.A.C.); choongsung.yoo@som.umaryland.edu (C.Y.); 2 Department of Anesthesiology, University of Maryland School of Medicine, Baltimore, MD 21201, USA; 3 Department of Physiology, University of Maryland School of Medicine, Baltimore, MD 21201, USA

**Keywords:** physical activity, maternal nutrition, offspring adipose, multi-omics

## Abstract

Embryogenesis is a critical process for which nutritional and metabolic signals act as informational cues that shape adipose tissue development and establish long-lasting metabolic health. Emerging evidence indicates that adipose tissue is not a passive energy storage but a developmentally and metabolically dynamic organ. Cellular composition, functional capacity, and plasticity of adipose are programmed early through coordinated transcriptional, epigenetics, and proteomics processes. Maternal environments in nutritional challenge, including overnutrition and malnutrition, influence adipocyte lineage commitment, depot-specific expansion, and metabolic functionality, predisposing offspring to divergent risks of obesity and metabolic disease. The future of perinatal adipose biology and genomics relies on integrating multi-omics approaches with an artificial intelligence (AI)-driven analytical perspective to resolve complex developmental processes and predict long-lasting metabolic health. Furthermore, the incorporation of sex-specific models is important, which will be essential for capturing biological heterogeneity and ensuring translational relevance. Together, these advance perspectives are predisposed to shift the field from descriptive associations toward predictive and preventive paradigms, reinterpreting metabolic disease risk as a modifiable consequence of early-life adipose programming rather than an inevitable outcome of later-life exposures.

## 1. Introduction

Maternal metabolic dysfunction and poor maternal diet habits, especially high-fat and high-caloric diets, are detrimental to both maternal metabolic health and fetal developmental programming, predisposing offspring to obesity and type 2 diabetes mellitus (T2DM) [1]. Physical activity and nutritional supplementation interventions have been recommended to combat pediatric obesity and metabolic dysfunction [2,3]. Nevertheless, the importance of maternal environmental changes for healthy mothers and their babies in the developmental origins of health and disease (DOHaD) perspective remains relatively underestimated. Physical activity is a direct and powerful tool to prevent and overcome obesity and metabolic dysfunction early in life, and the importance of exercise in pregnancy is gradually emerging and recognized as an early causal determinant of enhanced metabolic homeostasis in children [2,4]. Although “Exercise is Medicine” has been emphasized for fighting metabolic and chronic diseases, a better mechanistic understanding of why and how remains unclear at the molecular and genomic levels. The therapeutic potential of physical activity and nutritional supplementations mirroring exercise benefits can be due to its derived exercise hormones and biomarkers, which would allow patients who cannot do routine physical activity because of several reasons, such as pathophysiological symptoms, to partially get the beneficial effects of exercise.

Emerging pediatric obesity is not solely due to maternal obesity (MO), as the rates of childhood obesity are also increasing in mothers with various body mass abnormalities [5]. Therefore, we strongly believe that the existence of additional mechanisms is responsible for the increasing incident rates of childhood obesity. Similarly, maternal nutritional imbalance, including HFDs and low-protein diets, epigenetically programs offspring white fat by converging on DNA demethylation of the *Leptin* promoter, leading to *Leptin* gene expression [6,7,8,9,10]. On the other hand, maternal methyl-donor supplementation predisposes DNA methylation of the *Leptin* promoter, inhibiting its gene expression [11]. As for therapeutic potential, exercise in pregnancy has been introduced, which predisposes epigenetic modifications in offspring adipose tissue, regulating the expression of master energy regulators of the mitochondria, such as peroxisome proliferator-activated receptor gamma coactivator-1α (PGC-1α) [12]. Furthermore, maternal exercise (ME) also induces epigenetic modifications in offspring adipose tissue, showing an increase in the DNA demethylation of metabolically important gene promoters, including uncoupling protein 1 (*Ucp1*) and PR domain-containing protein 16 (*Prdm16*) [13,14].

In this review, we have synthesized evidence demonstrating that ME and nutritional interventions provide an instructive understanding that is genomically and epigenetically encoded in offspring adipose tissue. We focus on how these mechanistic signals regulate adipose tissue lineage commitment, mitochondrial biogenesis, and non-shivering thermogenic programming through transcriptional and epigenomic remodeling. Particularly, we have emphasized exercise-responsive endocrine factors, nutritional supplementations, and cross-cellular communications that mediate long-lasting effects on the metabolic function of adipose tissue in offspring. By integrating findings from animal models and emerging human clinical trials, this review aims to define critical knowledge gaps and outline future directions for leveraging maternal lifestyle interventions physically and nutritionally as a strategy to reprogram metabolic health across generations.

## 2. Adipose Tissue as a Memory Organ in Maternal–Fetal Medicine

Adipose tissue functions as a metabolic memory organ, which integrates maternal metabolic environments and reflects these signals in relatively stable molecular and cellular pathways that can influence downstream physiology [15,16]. This concept does not imply that adipose tissue itself directly transmits heritable information across generations, but rather that it serves as a dynamic interface through which maternal metabolic status is encoded and communicated through multiple mechanisms [15]. These mechanisms include endocrine signaling via adipokines and metabolites that influence placental and fetal physiology, local and systemic epigenetic remodeling within adipose progenitor and mature adipocytes, and indirect interactions with other regulatory systems, such as the placenta and neuroendocrine axes. In contrast, intergenerational inheritance is primarily mediated through germline epigenetic modifications in oocytes or sperm [17]. Therefore, in this review, adipose tissue as a metabolic memory organ refers to the capacity of maternal adipose tissue to integrate environmental inputs and maintain molecular signatures that shape fetal metabolic programming through endocrine and epigenetic pathways, while remaining mechanistically distinct from germline-based inheritance mechanisms.

Adipose tissue during pregnancy encodes maternal and offspring physiological phenomena, including glucose and insulin metabolism, mitochondrial stability, and other pregnancy complication-related pathophysiological conditions, such as gestational diabetes mellitus and preeclampsia [18,19,20,21]. Rather than solely acting as passive energy storage, maternal adipose tissue integrates pregnancy environmental conditions into molecular profiles that influence endocrine signaling for cross-organ communications [22,23]. Very interestingly, adipose tissue in mothers might be a key determinant organ of the intrauterine development in maternal–fetal medicine [24,25,26]. This is because maternal adipose-derived biological memory is inherited by the fetus through adipose-derived cytokines, called adipokines, including apelin, adiponectin, and leptin, and epigenetic factors, such as histone and DNA epigenetic modifications [10,27,28,29,30,31,32,33,34]. Taken together, fetal and offspring adipose tissues are programmed in accordance with maternal adipose memory, adopting transcriptional and epigenetic factors, which reflect maternal metabolic history, which then shapes adipocyte lineage allocation, non-shivering thermogenic capacity, and long-lasting metabolic flexibility in offspring.

From a maternal–fetal medicine perspective, maternal adipose memory interacting between mother and offspring can explain the reason why standard obstetric factors, including fetal growth and birth weight, often fail to capture long-lasting metabolic risk in offspring [35,36,37]. For example, obesity and respective pregnancy complications, such as gestational diabetes mellitus, induce adipose metabolic dysfunction, which can transmit maladaptive metabolic memory to the fetus through endocrine and metabolic pathways, leading to persistent changes in offspring adipose metabolic capacity [38,39,40]. On the other hand, physical activity during pregnancy can actively accumulate positive impacts in this adipose memory system, highlighting exercise as a precision medicine and intervention to improve fetal metabolic programming rather than barely controlling gestational weight gain [12,41,42]. Similarly, nutritional manipulations during pregnancy can also regulate the programming of adipose tissue development in offspring [43,44]. Together, maternal environmental challenges of exercise and nutrition can be therapeutic tools to transmit maternal adipose memory into the fetus for metabolic homeostasis.

## 3. Maternal Exercise and Nutritional Interventions in Offspring Adipose

Nutritional supplementations, commonly associated with molecular components in accordance with exercise adaptation, including proteins, essential fatty acids, micronutrients, and bioactive metabolites, influence maternal substrate utilization, adipokine secretion, and mitochondrial activity within adipose tissue [45,46,47,48]. Through these mechanisms, perinatal nutrition does not act independently, but rather conditions how maternal adipose tissue encodes and relays exercise-derived signals that ultimately program fetal and offspring adipose development [25,49]. Importantly, the interaction between exercise in pregnancy and sports nutrition has direct consequences for offspring adipose genomics and metabolic capacity [12,50]. Optimized nutritional environments may amplify exercise-induced remodeling of maternal adipose tissue, favoring the transmission of protective metabolic memory that enhances brown and beige adipocyte differentiation, mitochondrial biogenesis, and non-shivering thermogenesis in offspring. Emerging exercise-mimetic and metabolism-targeting nutrients, such as α-ketoglutarate and creatine, have garnered particular interest due to their roles in mitochondrial and energy metabolism, and epigenetic regulation, predisposing them as promising perinatal sports nutrition targets for shaping offspring adipose tissue development [51,52,53,54].

### 3.1. Maternal Exercise in Offspring Adipose Physiological Phenomena

ME during pregnancy improves offspring adipose tissue development and long-lasting metabolic health through multiple complementary mechanisms, including epigenetic alterations of adipogenic and thermogenic genes, normalization of adipose inflammatory signaling, improved insulin sensitivity and glucose metabolism, and sex-specific programming of white and brown adipose tissue [12,55,56,57,58,59,60,61,62,63,64,65,66], as summarized in Table 1. In detail, ME enhances brown adipose tissue (BAT) biogenesis and non-shivering thermogenesis, which is mediated by DNA demethylation of the *Prdm16* promoter and increased levels of maternal and fetal exercise-induced cytokines, called exerkines, such as apelin, protecting offspring from HFD-induced obesity [12]. ME also reduces adipose inflammation and metabolic dysfunction by increasing Serpin Family A Member 3C (SERPINA3C) in maternal adipose tissue and fetal circulation, which epigenetically reprograms fetal preadipocytes via the PI3K-TET1-KLF4 pathway [55]. Furthermore, ME mitigates hepatic and adipose metabolic dysfunction due to HFD-induced maternal obesity (MO) by normalizing metabolism-related gene expression and miRNAs [62], which is associated with reduced adipose inflammation, increased Adiponectin (gene: *Adipoq*) expression [57], an enhanced AMPK-PGC1α signaling pathway [59], and improved β-oxidation and lipogenesis in both dams and offspring [59,60]. ME also affects adipocyte morphology by reducing median adipocyte size, normalizing size distribution [58], and improving insulin, leptin, and triglyceride profiles, with some sex-specific effects [56,58,61]. Additionally, ME improves hypothalamic and neuroendocrine development, partially restoring αMSH and AgRP fiber density and Signal Transducer and Activator of Transcription 3 (STAT3)–ERK1/2 signaling axis disrupted by maternal HFD [63,66], whereas voluntary exercise reverses adverse outcomes associated with impaired maternal thermogenic capacity during pregnancy by improving offspring adipose tissue insulin sensitivity and endocrine function [64,65]. Together, these findings indicate that ME influences offspring adipose tissue through epigenetic, molecular, and endocrine pathways linked to metabolic homeostasis.

Human studies support the translational relevance of these findings, although effects are more modest. ME in late pregnancy, particularly moderate-to-vigorous physical activity (MVPA), is associated with slightly lower birth weight, reduced risk of being large-for-gestational-age (LGA), macrosomia, and ponderal index for infants, calculated as weight divided by height cubed (kg/m^3^), indicating healthier fetal adipose development [67]. Early-pregnancy maternal activity shows no direct association with offspring body composition at 24 months, though higher maternal patternicity modestly predicted higher child patternicity, and higher child activity was associated with lower body fat [68]. Moreover, ME also improves offspring glucose homeostasis and body composition, increasing lean mass and reducing fat mass in males, indicating sex-specific programming effects similar to animal models [64]. Overall, the available human data are consistent with preclinical studies and support a link between ME and offspring metabolic outcomes, although the magnitude of effect appears smaller than that observed in animal models.

ME represents a heterogeneous exposure rather than a single intervention, as treadmill running, voluntary wheel running, and moderate-to-vigorous physical activity differ in workload, physiological stress, and experimental reproducibility [69]. In animal studies, treadmill exercise provides greater control over exercise volume, frequency, duration, and precise intensity, whereas voluntary wheel running more closely reflects self-paced activity but introduces substantial variation in exercise doses and volumes between animals [70,71]. The gestational timing of exercise is also likely to influence biological outcomes, since early and late pregnancy may correspond to different windows of adipose tissue development and metabolic programming [4,72]. These methodological differences are important for interpreting mechanistic findings across studies and for understanding variability in reported outcomes.

### 3.2. Maternal Nutritional Interventions as Informational Signals

Nutritional intervention during pregnancy provides critical informative signals to the fetus, more than just serving as an energy source [73,74,75]. For instance, nutrients, including macronutrients, micronutrients, and bioactive metabolites, act as molecular signals that influence cellular differentiation [76,77], tissue development [78,79], and metabolic programming [80,81]. Developing adipose tissue in the fetus, including brown adipose tissue and adipocyte progenitor populations, is particularly sensitive to these signals, which can influence adipocyte maturation, depot formation, and later metabolic functionality [20,43,44]. Furthermore, maternal overnutrition or an HFD is associated with increased adiposity and impaired lipid metabolism in offspring [82,83,84,85]. When challenged with a short period of HFD, the offspring of obese dams exhibit suppressed hepatic circadian clock-related gene expression and reduced levels of key metabolic regulators, particularly peroxisome proliferator-activated receptor alpha (PPARα) and sirtuin 1 (SIRT1), with PPARα downregulation driven by decreased transcriptional activity, increased mRNA degradation [85]. Moreover, maternal HFD intake during pregnancy increased fetal lipid exposure by promoting maternal insulin resistance, inflammation, and elevated circulating free fatty acids, leading to early fetal fat accumulation and impaired development of metabolic tissues such as liver, adipose tissue, skeletal muscle, and pancreas [1,22,86].

On the other hand, maternal malnutrition impedes adipose tissue expansion and leads to long-lasting metabolic vulnerability [49,87,88]. In detail, maternal malnutrition predisposes adult male offspring to increased adiposity, as indicated by higher fat pad-to-body weight ratios, hyperleptinemia, an approximately 2-fold higher level of serum apelin, and white adipose tissue (WAT) transcriptional alterations, including up-regulated de novo lipogenic genes and dysregulated leptin/apelin signaling [89]. Under an HFD challenge, offspring from maternal malnutrition exhibited catch-up growth to match control body weight, transient hyperphagia, hypertrophied and hyperplastic gonadal adipocytes, increased STAT3 phosphorylation, and elevated plasma adiponectin [88]. However, there are some beneficial adaptive changes, such as enhanced leptin sensitivity and increased 11β-hydroxysteroid dehydrogenase type 2 (11β-HSD2) expression, which protect against additional fat accumulation [88]. Moreover, maternal protein restriction during pregnancy and lactation showed a similar phenotype in reducing birth weight and increasing visceral adiposity in adult offsprings [90]. In particular, in adult offspring, 650 transcripts in visceral WAT were differentially expressed, including the up-regulation of genes involved in adipocyte differentiation such as Peroxisome Proliferator-Activated Receptor Gamma (PPARγ) and CCAAT/Enhancer-Binding Protein Alpha (C/EBPα), carbohydrate and lipid metabolism, angiogenesis, and extracellular matrix remodeling [90]. These transcriptional changes indicate enhanced adipocyte number and activity, which contribute to increasing fat accumulation despite birth weight reduction. Interestingly, from this study, inflammation-related genes were largely down-regulated, which suggests that early-life programming promotes adipose expansion without inducing the concomitant inflammatory response [90]. Together, these findings listed in Table 2 support the concept that maternal diet functions as an instructive signal for the developmental programming of offspring adipose tissue.

## 4. Multi-Generational Epigenetic Mechanisms in Adipose Tissue

### 4.1. Epigenetic Factors for Exercise and Nutritional Interventions

**Figure 1:**
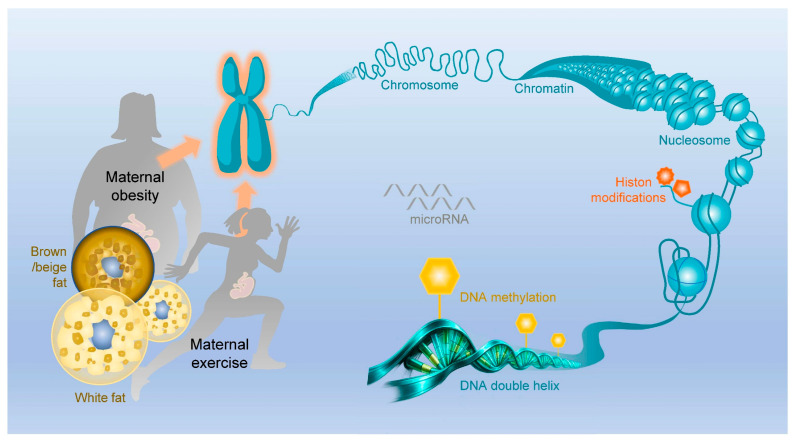
Schematic illustration of multi-layered transcription-to-translation by various epigenetic modifications. Maternal nutrition and physical activity predispose the regulation of epigenetic factors, including DNA (de)methylation, histone (de)demethylation/(de)acetylation, chromatin modification, as well as microRNAs (miRNAs) in perinatal brown/beige and white adipose tissues.

Epigenetics describes biological regulations in which gene expression is controlled independently of DNA sequence, allowing the same genome to generate diverse and stable cellular phenotypes [96]. Rather than encoding information in nucleotide changes, epigenetic regulation operates by modulating chromatin organization and transcriptional permissiveness through DNA methylation and histone- and chromatin-associated modifications, as well as non-coding (nc)RNAs (Figure 1). These regulations are inherently responsive to physiological and environmental inputs, enabling signals of nutrient, endocrine, inflammation, and physical activity to be translated into persistent changes in gene expression profiling [97,98]. Because epigenetic factors can be maintained through cell division but remain reversible, they provide a mechanism for both biological memory and adaptability [99]. Thus, epigenetics has a key role as a dynamic interface between genotype and environment, which shapes developmental programming, metabolic function, and long-lasting disease risk [100,101].

### 4.2. Epigenetic Modifications of Offspring Adipose Tissue

Emerging evidence suggests that the impact of maternal nutrition and/or obesity-related metabolic stress may extend beyond the immediate offspring to subsequent generations, a process mediated in part through germline epigenetic modifications [102,103,104], leading to regulating the development of adipose tissue, a process termed nutritional encoding [49,105]. Specifically, several studies demonstrate that nutritional manipulation alters DNA and chromatin (de)methylation in oocytes and sperm, which transmits metabolic memory and information to the next generation [106,107,108]. Of note, several animal studies elucidated that epigenic changes in sperm and eggs, such as H3K4me3 (an activator) and H3K27me3 (an inhibitor), normally activate genes in relation to longevity and metabolism by insulin/IGF-1 and mTOR/AMPK pathways, which affects lifespan and metabolism in offspring, suggesting that these epigenetic changes are inherited [104,106]. On the other hand, dietary manipulation also exerts beneficial epigenetic changes [91]. In the yellow agouti mouse model, maternal supplementation with methyl-donor nutrients restores DNA methylation at the agouti locus, resulting in reduced adiposity and improved metabolic health in offspring, with DNA methylation-based epigenetic modifications that persist across generations [91]. Moreover, the mid-gestational maternal supplementation with methyl-donors showed modification of the germline epigenetic factors of a silenced Avy allele [109]. F1 females exposed in utero produced F2 offspring that also displayed a shift toward the pseudo-agouti phenotype, which indicates that the beneficial epigenetic changes, such as increased DNA methylation at the Avy locus, persisted through gametogenesis and embryonic development, even without additional dietary supplementation [91,109]. These studies highlight how maternal diet induces heritable improvements in metabolism and adiposity across multiple generations.

DNA methylation, histone modifications, and non-coding RNA expression are key mechanisms by which maternal diet shapes the adipose epigenome [105,110,111]. These epigenomic changes regulate gene profiles involved in lipid storage, adipogenesis, and endocrine function, effectively imprinting adipose tissue in their offspring. For instance, Lin et al., 2021 [10] reported that when pregnant dams were exposed to an HFD, offspring showed persistently increased *leptin* expression in fetal and postnatal adipose tissues despite normalization of their body weight later in life, indicating long-lasting reprogramming of adipose endocrine function. This was caused by the hypomethylation of the *leptin* promoter and increased ten-eleven-translocation 1 (TET1) expression in adipose tissue, and these changes were recapitulated when fetal mesenchymal stem cells were treated with palmitic acid during adipogenic differentiation [10]. Similarly, in another metabolic tissue, skeletal muscle, Laker et al., 2014 [112] reported that when dams were fed an HFD, offspring exhibited hypermethylation of the peroxisome proliferator-activated receptor γ coactivator-1α (*Pgc1a*) promoter in skeletal muscle from birth through 12 months old, accompanied by a trend toward reduced *Pgc1a* expression, indicating persistent epigenetic repression of a key metabolic regulator gene. However, through ME, the hypermethylation of the *Pgc1a* promoter was prevented, and the gene expression was restored, demonstrating that early-life interventions reverse epigenetic imprinting of metabolic genes induced by maternal obesogenic diet [112]. Moreover, maternal obesity reduces PPARα expression by epigenetic remodeling at its promoter, marked by altered histone H3 lysine 4 trimethylation (H3K4me3, active form) and histone H3 lysine 27 trimethylation (H3K27me3, repressive form) [85]. Lastly, when mouse dams were fed an HFD before and during pregnancy and lactation, offspring displayed impaired BAT structure, elevated DNA methylation of thermogenic gene Cox7a1 and fatty acid oxidation-related genes, such as *Acaa2* and *Acsl1*, with reduced expression of these genes at 16 weeks of age, indicating that excess intra-uterine energy intake epigenetically reprograms BAT metabolism in offspring [92]. These studies indicate that maternal lipid excess directly imprints adipose progenitors through stable epigenetic modification.

Similarly, paternal nutritional excess or imbalance has been shown to influence inflammatory and metabolic gene expression through epigenetic regulation, suggesting that germline-encoded responses to the nutritional environment may predispose subsequent generations to altered adipose development and metabolic dysfunction [81,113,114,115]. In a Wistar rat model, HFD-fed fathers transmitted an obesogenic phenotype to their offspring, with associated hypermethylation of the Proopiomelanocortin (*Pomc*) promoter in sperm and in the hypothalamic arcuate nucleus of weanling offspring [113]. These epigenetic changes were linked to increased DNMT3B expression and affected genes involved in neuronal development, suggesting that paternal hypermethylation at specific CpG islands within the *Pomc* promoter mediates inter-generational transmission of obesity.

**Figure 2:**
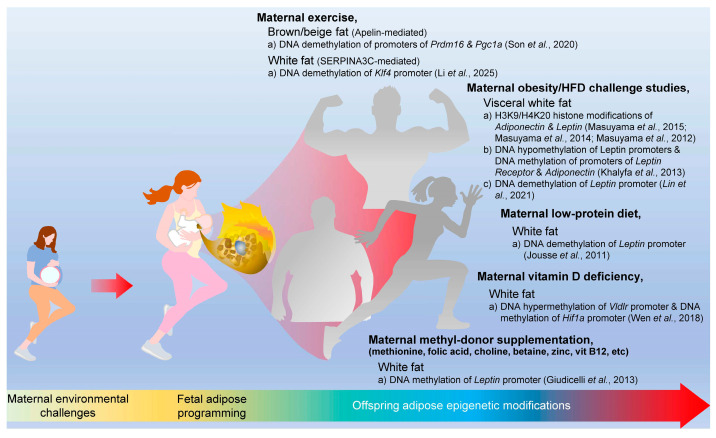
Maternal environments in nutrition and exercise program offspring adipose epigenomics. Maternal exercise induces either apelin- or serpina3c-mediated DNA demethylation in offspring brown/beige and white fat. Maternal obesity and high-fat diet further regulate white fat histone and DNA methylation of *Adiponectin* and *Leptin* promoters. Other maternal environments, including low-protein diet, vitamin D, and methyl-donor supplementation, also regulate DNA methylation in offspring white fat [6,7,8,9,11,12,55,93,95,97].

Recent studies further suggest that maternal metabolic environments influence the adipose epigenome at a genome-wide scale rather than through isolated gene-specific mechanisms [49,116,117]. Epigenome-wide methylation analyses have demonstrated that maternal obesity and high-fat diet exposure induce widespread remodeling of histone or DNA methylation patterns in metabolic tissues, including adipose and liver, affecting gene networks involved in lipid metabolism, mitochondrial function, and inflammatory signaling [118,119,120]. These findings indicate that developmental programming operates through the coordinated regulation of metabolic pathways rather than single-gene effects. Emerging single-cell transcriptomic and epigenomic approaches are beginning to reveal cell-type-specific regulatory landscapes within adipose tissue, which provides further insight into how maternal environmental challenges shape adipocyte lineage commitment and metabolic function [121,122]. Expanding these integrative epigenomic analyses will be essential for understanding the system-level architecture of perinatal metabolic programming. Together, these epigenetics studies indicate that both maternal and paternal nutrition induce heritable epigenetic changes that influence metabolism, adiposity, and disease risk across multiple generations, highlighting the critical role of nutritional environments in shaping long-lasting health outcomes in metabolism (Figure 2).

## 5. Translational Relevance of Adipose in Pregnancy

Establishing translationally relevant evidence linking maternal nutrition and lifestyle during pregnancy to offspring adipose development is essential for preventing lifelong metabolic disease [123,124]. Adipose tissue development is programmed during early life [123,124], but the direct interrogation of fetal tissues in humans remains limited, creating a critical gap between experimental discoveries and clinical application. Addressing this gap requires an integrative investigation that links human longitudinal data, accessible biomarkers, and pregnancy-compatible interventions. In this review, maternal diet/nutrition and physical activity represent uniquely translatable targets, as they are easily accessible, safe, and scalable within routine prenatal care. Advancing translationally grounded research in this area has the potential to inform evidence-based interventions that optimize maternal metabolism, support placental function, and durably improve offspring metabolic health across the life.

### 5.1. Birth Cohorts and Longitudinal Studies

Longitudinal birth cohort studies provide critical insight into the impact of maternal nutrition on offspring adipose tissue development [125,126] and metabolic health [127,128]. From a cohort study of 2854 birthing parent–child dyads, higher prenatal healthy eating index [79] scores were associated with birth weight and growth kinetics within reference ranges [129]. Moreover, from a prospective longitudinal birth cohort in Project Viva of 1060 mother–child dyads, higher prenatal dietary inflammatory index scores were associated with greater overall and central adiposity accrual from early childhood through adolescence, with stronger effects observed in the presence of maternal psychosocial stress [130]. Consistently, in a larger Project Viva analysis of 1459 mother–child dyads, poorer maternal diet quality during pregnancy, which is characterized by higher dietary inflammatory potential and lower adherence to a Mediterranean-style diet, was associated with accelerated childhood BMI *Z*-score gain and higher adiposity in later childhood and adolescence, independent of key maternal and postnatal confounders [131]. The observed associations between maternal dietary inflammatory potential, Mediterranean diet adherence, and BMI *Z*-score gain of offspring may reflect early-life programming through inflammatory and metabolic pathways. Notably, these effects appear across multiple sensitive periods of growth from infancy to adolescence, underscoring the importance of consistent prenatal nutrition. While findings align with other longitudinal birth cohorts, limitations such as self-reported dietary intake and demographic homogeneity should be considered. In conclusion, these results highlight the potential for prenatal dietary interventions to influence long-lasting metabolic health in offspring.

### 5.2. Challenges in Clinical Epigenetic Studies

Clinical studies face several challenges, including securing large enough sample sizes, especially in longitudinal cohorts, tissue accessibility, temporal variability in adipose epigenetic marks, and confounding environmental factors. Sampling fetal or neonatal adipose tissue is invasive and limited, necessitating the use of surrogate tissues or circulating biomarkers [132,133]. Furthermore, epigenetic modifications are dynamic and influenced by the postnatal environment, complicating causal inference [134,135]. Despite these limitations, advances in minimally invasive sampling techniques [136,137], single-cell epigenomics [138,139], and integrative computational models [140,141] are improving our ability to translate mechanistic insights into clinical contexts.

### 5.3. Translational Interventions in Pregnancy

Understanding the epigenetic and genomic programming of adipose tissue enables the design of targeted interventions to optimize maternal and offspring metabolic health. Both dietary- and physical activity-based interventions have shown potential to modulate fetal adipose development through effects on maternal metabolism, inflammation, and epigenetic regulation [112,142,143]. Several studies have already pointed out that poor maternal intake, in terms of both quantity and quality, may affect neonatal adipose accretion [125,144,145]. Moreover, even in women who are not at risk of malnutrition, micronutrient intake was associated with neonatal anthropometry [146]. Neonatal abdominal circumference, which is an indicator of central adiposity, was positively associated with third-trimester retinol intake and inversely associated with vitamin E and selenium intake [146]. Waist-to-length ratio was negatively associated with third-trimester magnesium intake, and the subscapular-to-triceps skinfold ratio, reflecting relative central fat distribution, was negatively associated with first-trimester selenium intake [146]. Similarly, ME during pregnancy has emerged as a non-pharmacological intervention capable of improving insulin sensitivity, reducing systemic inflammation, and influencing placental and fetal metabolic signaling, with emerging evidence suggesting epigenetic modulation of genes involved in adipogenesis and energy homeostasis [12,147,148,149]. ME during pregnancy improves placental oxidative metabolism and function by enhancing mitochondrial biogenesis, vascularization, and nutrient transport, potentially through exerkine- and placentokine-mediated signaling such as apelin, irisin, adiponectin, superoxide dismutase (*SOD*) 3, thereby supporting fetal development and long-term metabolic health of the offspring [147,148,149]. Moreover, ME elevates the exerkine apelin, activating AMPK-dependent metabolic and epigenetic pathways that promote PRDM16-mediated brown and beige adipose tissue development, enhance non-shivering thermogenesis, and protect offspring from obesity and metabolic dysfunction [12]. Maternal aerobic exercise during pregnancy helps control gestational weight gain, improves maternal metabolic health, and benefits fetal and neonatal outcomes, including lower neonatal adiposity and healthier birth weight through growth- and metabolism-related genes, such as Insulin-like Growth Factor 2 (*Igf2*), *leptin*, and *Pgc1a*. Altogether, these findings highlight ME as a safe and effective strategy to optimize gestational weight gain, enhance maternal metabolism, and epigenetically program offspring adipose tissue for long-lasting metabolic health.

## 6. The Future of Perinatal Adipose Biology and Genomics

The future of perinatal adipose biology and genomics will be shaped by three overarching and complementary components. First, multi-omics perspectives and artificial intelligence (AI)-driven analysis will enable an integrated understanding of how perinatal signals program adipose tissue across the lifespan. Of note, AI and machine learning [150] applications will be essential for identifying latent patterns, developmental signatures, and predictive markers that are not apparent through conventional analyses, ultimately supporting early risk stratification and precision preventive strategies [151]. Second, sex-specific biological determination will become central to advancing biological relevance and translational impact. Accounting for sex-dependent hormone–adipose interactions during the perinatal period is therefore critical for accurately defining developmental programming mechanisms and for improving the translational relevance of perinatal adipose genomics. Last, although current mechanistic evidence emphasizes exercise-responsive endocrine and epigenetic pathways, additional independent mechanisms, including placenta-specific adaptations, broader myokine networks, and fetal neuroendocrine modulation, would contribute to ME-mediated adipose programming and its respective systematic investigation.

### 6.1. Integrative Multi-Omics Approaches and AI-Driven Analysis

Integrative multi-omics approaches provide a powerful tool for screening target biomarkers by capturing regulatory expression across multiple biological systems from gene transcription to protein translation. RNA sequencing (RNA-seq) has been widely used in adipose to identify transcriptional signatures predictive of insulin resistance and mitochondrial dysfunction [152]. DNA methylation sequencing (methyl-seq) in adipose tissue has revealed dynamic epigenetic alterations in obesity that are potentially associated with a therapeutic biomarker for metabolic dysfunction [153]. Proteomics has further strengthened biomarker discovery by confirming whether transcriptional and epigenetic changes are translated into functional protein coding, as demonstrated in an adipose study where coordinated RNA and protein signatures outperformed single-omics predictors [154,155]. Applying this layered strategy, combining RNA-seq, methyl-seq, and proteomics to perinatal adipose tissue, allows for the prioritization of biomarkers that are transcriptionally active, epigenetically programmed, and functionally executed, increasing robustness and translational potential. AI-driven analysis extends these discoveries by linking molecular biomarkers to tissue-level structures and early phenotypic changes in perinatology [156]. On the other hand, there is a lack of understanding of multi-omics analysis in perinatal adipose tissue. In different tissue and pathology systems, such as oncology and neurodegenerative research, ML models integrating transcriptomic data with histological features have enabled early disease classification before clinical symptoms emerge [157,158]. Similarly, in musculoskeletal and cardiovascular systems, AI-based analysis of MRI and X-ray images has been used to predict tissue degeneration and metabolic dysfunction when combined with molecular profiles [159,160]. Translating this paradigm to perinatal adipose biology, AI can integrate candidate biomarkers identified through multi-omics with quantitative histology and imaging-derived features to support early diagnosis and biomarker targeting. This approach emphasizes AI as a critical bridge between molecular discovery and clinically actionable prediction, even when direct disease phenotypes are not yet apparent.

Recent technological advances further expand the resolution at which adipose programming can be investigated. For instance, single-cell RNA sequencing (scRNA-seq) has begun to reveal previously unrecognized heterogeneity among adipocyte progenitors, immune cells, and stromal populations within adipose tissue, which enables identification of cell-type-specific transcriptional responses to metabolic stress and developmental status [161,162]. Spatial transcriptomics provides an additional strategy by preserving tissue architecture, which allows investigators to map gene expression patterns within distinct adipose tissues and to understand how cellular interactions shape thermogenic and metabolic functions [163,164]. In parallel, epigenome-wide association studies (EWAS) are increasingly used to identify DNA methylation signatures associated with maternal metabolic environments and offspring metabolic risk [49,116,117,118,119,120]. Integrating these high-resolution datasets with AI-driven analytical frameworks will enable systematic identification of regulatory networks and predictive biomarkers underlying perinatal adipose programming, strengthening the translational potential of multi-omics approaches.

### 6.2. Sex-Specific Biological Considerations

Future advances in adipose biology will require explicit integration of sex-specific biological perspectives, as males and females are exposed to fundamentally different hormonal environments across development and throughout life [165]. Sex hormones such as estrogens and androgens exert powerful regulatory effects on adipose tissue differentiation, distribution, and endocrine function [150,166,167], whereas adipose tissue itself actively produces and metabolizes hormones that shape systemic physiology [168]. During the perinatal period, these bidirectional hormone-adipose interactions are established early and can differentially program adipose developmental programming in males and females, leading to persistent divergence in metabolic outcomes [169]. Despite this, many developmental and genomic studies of adipose tissue have historically pooled sexes or focused on a single sex, limiting mechanistic resolution and translational relevance. Future research should, therefore, adopt sex-stratified experimental designs and analytical strategies to capture hormone-dependent adipose programming, recognize sex-specific vulnerability and resilience mechanisms, and ultimately enable more accurate prediction and intervention strategies tailored to male and female metabolic biology.

### 6.3. Additional Mechanisms of Maternal Exercise Programming

Importantly, while current mechanistic evidence has largely emphasized exercise-responsive endocrine mediators and epigenetic remodeling pathways, additional independent mechanisms are likely to contribute to ME-mediated programming of offspring adipose tissue but remain insufficiently characterized. For example, exercise-induced placental adaptations independent of circulating exerkines, including changed placental mitochondrial biogenesis, vascular remodeling, and nutrient transporter activities, may directly influence fetal substrate exposure and adipose lineage allocation. In parallel, myokines beyond apelin, such as irisin and IL-6, may participate in fetal tissue priming through endocrine or paracrine mechanisms, although their causal roles in perinatal adipose programming remain to be rigorously defined. Furthermore, ME may modulate fetal neuroendocrine development, which indirectly shapes BAT thermogenesis through central regulatory pathways. These mechanisms represent critical knowledge gaps using integrated placental, fetal, and neuro-adipose axis approaches.

## 7. Limitations and Translational Challenges in Animal and Human Studies

Animal models have been instrumental in establishing mechanistic links between ME, nutrition, and offspring adipose programming [12,55,56,57,58,59]. However, important biological and translational limitations remain unclear. Rodents differ from humans in gestational duration, placental architecture, developmental timing of adipose depots, thermogenic reliance, and lifespan metabolic transition [170,171]. BAT is proportionally more abundant and functionally dominant in rodents, and non-shivering thermogenesis contributes more substantially to systemic energy balance than in adult humans [172]. These differences complicate direct translation of findings related to BAT activation, PRDM16 signaling, and mitochondrial remodeling. Moreover, experimental paradigms such as HFD exposure often represent uniform and extreme metabolic challenges that may not accurately reflect the complexity of human nutritional environments. Laboratory animals are also genetically homogeneous and maintained under controlled conditions, which improves internal validity but limits generalizability. In addition, epigenetic inheritance observed across multiple generations in rodents may not fully translate to humans due to species-specific differences in germline epigenetic reprogramming.

Human observational and interventional studies present additional challenges. Definitions and measurements of maternal physical activity vary widely, and many cohort studies rely on self-reported questionnaires that are susceptible to recall bias and misclassification. Objective measures such as accelerometry or heart-rate monitoring, together with standardized intensity classifications aligned with prenatal exercise guidelines, would improve interpretability. Other sources of variability include maternal metabolic conditions prior to conception, gestational weight gain patterns, genetic background, and inflammatory conditions. Furthermore, epigenetic analyses often rely on accessible tissues such as cord blood or placenta, which may not fully represent adipose-specific programming.

Finally, effect sizes reported across studies vary considerably depending on experimental design, exposure timing, and population characteristics. In human cohorts, associations are often modest and may be influenced by postnatal environmental factors such as nutrition, lifestyle behaviors, and socioeconomic conditions. Reproducibility across cohorts and analytical platforms remains an ongoing challenge for epigenetic and multi-omics studies. Together, these considerations highlight the need for standardized experimental approaches, independent validation, and integrative human studies to better define the magnitude and persistence of perinatal adipose programming.

## 8. Conclusions

In conclusion, this review underscores perinatal adipose biology as a central determinant through which nutritional and exercise-derived signals are integrated and encoded to shape offspring metabolic health across generations. Maternal nutrition and physical activity act as interventional tools that converge on transcriptional, epigenetic, and proteomic regulations of adipose development, establishing molecular programs that persist beyond early life and influence long-lasting metabolic health. Advances in multi-omics and AI-driven analyses provide the necessary strategy to decode this complex integration. Furthermore, sex-specific biological considerations are essential for accurately capturing hormone–adipose interactions that modulate these effects. Taken together, a cross-generational perspective that unifies exercise and nutritional encoding within adipose genomics is a pivot to prevent metabolic disease as a developmental and inter-generational process, rather than a late-stage therapeutic challenge.

## Figures and Tables

**Table 1:** Maternal exercise effects on offspring adipose-related outcomes.

Ref	Subject	Exercise Type	Time Point	Offspring Adipose-Related Outcome
[12]	C57BL/6J mouse (n = 6)	Treadmill running	Pregnancy	- Apelin-mediated signaling (↑) - Brown/beige adipogenesis (↑) & NST (↑) - by *Prdm16* promoter DNA demethylation (↑)
[55]	C57BL/6J mouse + HFD (n = 6)	Treadmill running	Pregnancy	- SERPINA3C-mediated signaling (↑) - White adipose inflammation (↓) - by *Klf4* promoter DNA demethylation (↑)
[56]	Wistar rat + LPD (n = 6)	Treadmill exercise	Offspring: adulthood	- vWAT leptin (↓)
[57]	C57BL/6J mouse + OB (n ≥ 7)	Voluntary wheel running	Pre-pregnancy + gestation	- eWAT adiposity and inflammation (↓)
[58]	Albino Wistar rat + OB (n = 8)	Voluntary wheel running	Mother: pregnancy + lactation Offspring: P50–P110	- Adipocyte size (↓) & distribution (↓)
[59]	C57BL/6N mouse + OB (n ≥ 9)	Voluntary wheel running	Pre-pregnancy + gestation	- egWAT adipose accumulation (↓) & hepatic steatosis (↓) - by AMPK–PGC-1α axis (↑)
[61]	Sprague-Dawley rat + OB (n = 12 or 16)	Voluntary wheel running	Pregnancy	- Adipose GLUT4 activity (↑) & inflammatory signaling (↓) - Systemic metabolic outcomes (glucose ↓, TG ↓)
[62]	C57BL/6J mouse + HFD (n = 6)	Voluntary wheel running	Pre-pregnancy + gestation	- sWAT/VAT mass (↓) - Systemic metabolic outcomes (glucose tolerance ↑)
[63]	C57BL/6N mouse + HFD (n = 7–9)	Voluntary wheel running	Pregnancy	- WAT IL-6 signaling normalization (↑) - Systemic metabolic outcomes (glucose tolerance ↑, serum leptin ↓)
[64]	ICR mouse (n = 18 or 20)	Voluntary wheel running	Pre-pregnancy + gestation + lactation	- Adipose mass (↓) - Systemic metabolic outcomes (glucose tolerance ↑)
[66]	Sprague-Dawley rat + HFD (n = 6 or 7)	Voluntary wheel running	Pregnancy + lactation	- Adipose mass (↓)

Non-shivering thermogenesis, NST; subcutaneous white adipose tissue, sWAT; visceral white adipose tissue, vWAT; epididymal white adipose tissue, eWAT; epigonadal white adipose tissue, egWAT; postnatal day, P; low-protein diet, LPD; triglyceride, TG; Institute for Cancer Research, ICR; high-fat diet, HFD. ↑ (increase) and ↓ (decrease).

**Table 2:** Maternal nutritional intervention effects on offspring adipose-related outcomes.

Ref	Subject	Nutritional Exposure	Time Point	Offspring Adipose-Related Outcome
[6]	ICR mouse + HFD (n = 12)	HFD	Pre-pregnancy + gestation + multigeneration	- Adipose mass (↑) & vWAT leptin (↑) and adiponectin (↓) - by H3K9 acetylation of adiponectin (↓) & H4K20 monomethylation of leptin (↑)
[7]	ICR mouse + HFD (n = 6)	HFD	Pregnancy	- Serum leptin (↑) and adiponectin (↓) & WAT leptin (↑) and adiponectin (↓) - Systemic metabolic outcomes (blood pressure ↑, glucose tolerance ↓, TG ↑) - by H3K9ac (↓) and H3K9me (↑) histone modification of adiponectin promoter & H4K20me1 (↑) of leptin promoter
[8]	C57BL/6J mouse + HFD (n = 36)	HFD	Late pregnancy + lactation	- Adiposity (vWAT ↑ and sWAT ↑) & postnatal weight gain (↑) - vWAT leptin (↑), adiponectin (↓), and leptin receptor (↓) - by vWAT DNA methylation of Adiponectin (↑), leptin receptor (↑), and leptin (↓) - Systemic metabolic outcomes (insulin resistance ↓, glucose tolerance ↓)
[9]	BALB/C mouse + LPD (n = 10)	LPD	Pregnancy + lactation	- Birth weight (↓) & fat mass (↓) - Plasma leptin (↓) and WAT leptin (↓)
[10]	Sprague-Dawley rat + HFD (n = 20)	HFD	Pregnancy	- Fetal & early postnatal adiposity (↑) & sustained hyperleptinemia (↑) & adult hypertension (↑) - by adipose leptin promoter hypomethylation (↑)
[11]	Sprague-Dawley rat + MeDo + PRD (n = 8)	MeDo + PRD	Pre-pregnancy + gestation + lactation	- Adipose-derived leptin secretion (↓) & plasma leptin (↓) - by DNA methylation of leptin promoter (↑) - Systemic metabolic outcomes (TG ↓, cholesterol ↓)
[56]	Wistar rat + LPD (n = 6)	LPD	Pregnancy	VAT leptin (↑)
[85]	Sprague-Dawley rat + OB (n = 15)	Overnutrition / HFD	Pregnancy	Hyperinsulinemia (↑)
[87]	Wistar rat + FR (n = 25)	30% of food intake (restriction)	Pregnancy	- sWAT and rWAT adipocyte size (↑) - Systemic metabolic outcomes (insulin resistance ↓)
[90]	Wistar rat + LPD (n = 6)	LPD	Pregnancy + lactation	- Birth weight (↓) & vWAT mass (↑) - vWAT PPARγ (↑), C/EBPα (↑), lipid metabolism (↑), angiogenesis (↑), ECM remodeling (↑) & inflammatory gene expression (↓)
[91]	A^vy^ mouse + MeDo (n = 29, 33)	MeDo	Pre-pregnancy + gestation	- Transgenerational body weight (↑)
[92]	C57BL/6J mouse + HFD (n = 16)	HFD	Pre-pregnancy + gestation + lactation	- BAT adiposity (↑) - by hypermethylation of Acaa2 (↑), Acsl1 (↑), and Cox7a1 (↑)
[93]	ICR mouse + HFD (n = 6)	HFD	Pregnancy + lactation	- Body weight (↑) and fat mass (↑) & vWAT leptin (↑) and adiponectin (↓) & serum leptin (↑) and adiponectin (↓) - Systemic metabolic outcomes (glucose tolerance ↓, TG ↑)
[94]	Wistar rat + FR (n = 12)	Mother: 30% of food intake (restriction) Offspring: HFD	Pregnancy FR + postnatal HFD	- Adipose hyperleptinemia and hypercorticosteronemia (↑) - Systemic metabolic outcomes (glucose tolerance ↓, TG ↑)
[95]	Sprague-Dawley rat + VitD deficiency (n = 21–22)	Vitamin D deficiency	Pregnancy	- Adiposity (↑) & dyslipidemia (↑) - WAT DNA methylation of Vldlr promoter (↓) and Hif1a promoter (↑)

Visceral white adipose tissue, vWAT; subcutaneous white adipose tissue, sWAT; food restriction, FR; retroperitoneal white adipose tissue, rWAT; low-protein diet, LPD; extracellular matrix, ECM; methyl-donor, MeDo; protein restriction diet, PRD; vitamin D, VitD; high-fat diet, HFD. ↑ (increase) and ↓ (decrease).

## Data Availability

No new data were created or analyzed in this study. Data sharing is not applicable to this article.
